# Green remediation of lead (pb) from Pb-toxic soil by combined use of silicon nanomaterials and leguminous *Lens culinaris* L. plants

**DOI:** 10.1038/s41598-025-88759-x

**Published:** 2025-02-05

**Authors:** Tayyaba Naz, Muhammad Mazhar Iqbal, Bilal Raza, Muhammad Asad Mubeen, Muhammad Ather Nadeem, Abdullah Ahmed Al-Ghamdi, Mohamed S Elshikh, Muhammad Rizwan, Rashid Iqbal

**Affiliations:** 1 Institute of Soil and Environmental Sciences, University of Agriculture, Faisalabad, 38040 Pakistan; 2 Saline Agriculture Research Centre, University of Agriculture, Faisalabad, 38040 Pakistan; 3 Department of Soil and Environmental Sciences, College of Agriculture, University of Sargodha, Sargodha, 40100 Pakistan; 4 Lincoln Institute of Agriculture Food Technology, College of Health and Science, University of Lincoln, Riseholme Park, Lincoln, LN2 2LG UK; 5 Department of Agronomy, College of Agriculture, University of Sargodha, Sargodha, 40100 Pakistan; 6 Department of Botany and Microbiology, College of Science, King Saud University, P.O. 2455, Riyadh, 11451 Saudi Arabia; 7 Institute of Crop Science and Resource Conservation (INRES), University of Bonn, 53115 Bonn, Germany; 8 Department of Agronomy, Faculty of Agriculture and Environment, The Islamia University of Bahawalpur, Bahawalpur, 63100 Pakistan; 9 Department of Life Sciences, Western Caspian University, Baku, Azerbaijan

**Keywords:** Legumes, Nano-silicon, Pb toxicity, Silicon, Total chlorophyll contents, Plant sciences, Environmental sciences

## Abstract

Lead (Pb) toxicity is a major issue due to anthropogenic activities that is faced by farmers nowadays which inhibits plant growth and decreases crop yields. From contaminated soils, Pb absorbed by the plants and then ultimately enters into the food chain. Silicon (Si) can reduce Pb availability to plants and can be helpful in Pb immobilization in the soils. Moreover, Si in its nano-form, is expected to augment the beneficial attributes of applied Si. However, very little is known regarding the prospects of nano-Si application and leguminous lentil for alleviating the effects of Pb stress. To assess the effectiveness of bulk Si and nano-Si for reducing Pb toxicity and improving the yield of lentils, a pot study was conducted. Lentil variety Punjab Masoor 2020 was examined under normal and Pb toxic conditions as affected by applied Si and nano-Si. There were eight treatments comprised of different combinations of Si at 100 and 200 mg Si kg^− 1^ soil, and nano-Si at 125 mg kg^− 1^ soil, which were tested against Pb at 500 mg kg^− 1^ soil. A completely randomized design with factorial arrangements was applied along with three replications each. The result showed that Pb toxicity reduced the plant growth, yield, total chlorophyll contents, membrane stability index, relative water content, shoot fresh weight and dry weights of lentil. Whereas Si and nano-Si lessened the negative effect of Pb toxicity by significantly reducing its concentration in plant roots and shoot, and improved agro-physiological traits of lentil in normal and Pb-toxic soil conditions. In soil spiked with 500 mg kg^− 1^ Pb, the application of 100 and 200 mg bulk Si per kg of soil and 125 mg kg^− 1^ nano-Si reduced the Pb concentration in shoot by 31, 62 and 84% respectively over controls. In squat, the application of nano-Si most significantly (*p* ≤ 0.05) reduced the root and shoot Pb concentration in lentil.

## Introduction

Lead (Pb) is considered a toxic heavy metal, which is one of the main reasons for environmental pollution. Anthropogenic activities like the production of industrial waste, gasoline, fertilizers, and explosives cause Pb to appear in environment. Levels of Pb in soils affected with Pb vary from 400 to 800 mg kg^− 1^ soil, while in industrialized areas 1000 mg Pb kg^− 1^ soil is reported^1^. It is estimated that there is 10% contribution of Pb in environmental pollution occasioning from heavy metals. Lead is an extremely contagious heavy metal for living organisms. Lead consists of 0.002% of Earth’s crust. Lead pollutes the environment through anthropogenic activities and due to natural resources like volcanic eruptions which majorly contribute to environmental Pb contamination^2^.

Lead affects humans in various ways including disruptions in hemoglobin synthesis, the development of kidney problems and high blood pressure in adults, and negative impacts on the growth of children^3^. Lead toxicity leads to delay the plant growth and induces oxidative stress in plants. Lead-treated and untreated plants show significant morphological variations^4^. Radicle emergence is inhibited by Pb exposure in plants via interfering with the protein and starch metabolic pathways, and Pb changes proteases, amylases, acid phosphatases and the amounts of proteins and carbohydrates, as well as enzymatic function of plant cells^5^.

Silicon (Si) is a beneficial element because of its substantial involvement in giving several advantages for plant development, specifically in stressed conditions^6^. Silicon has several critical roles in crop growth including acceleration of plant growth, production, and quality, photosynthesis, nitrogen fixation, and provides plants with the ability to withstand different stresses like high temperatures, ultraviolet rays, metal toxic effect, nutritional deficiencies, drought, salinity, pathogens and fungus attack^7^. Silicon application can induce tolerance in plants against toxic metals by sequestering the metal within the cell wall of the root, thereby preventing its movement into the cytoplasm^8^. This is achieved by Si’s ability to form covalent bonds with metals, leading to the creation of metal-silicate complexes that effectively mitigate metal toxicity^9^.

By creating nanotechnology-based fertilizers, agricultural crops growth and yield may be improved. The unusual mesoporous properties of nano-silica, a special kind of Si-derived nanomaterial, make it an ideal nano-carrier for various chemicals and compounds that enhance agricultural yields^10^. The Si nanoparticles, synonymously nano-Si, are ingested by plants through their roots, which undergo a transformation into polymers prior to their transfer and preservation within the shoot tissues^11^. In addition, nano-Si are transported by active routes into the plant xylem^12^. Physiological characteristics of the nano-Si particles allow them to permeate plants and alter metabolism^13^. Nano-Si have been reported to enhance plant resilience against both biotic and abiotic stressors, thereby reducing their adverse impacts^14^.

Lentil *(Lens culinaris* L.) is a kind of edible legume and belongs to family fabaceae. The plant is characterized as an annual with seeds that have a lens-like shape. The plant reaches a height of approximately 40 cm (16 in), and its seeds develop within pods containing two seeds each. Lentils are distinguished for their high protein content, starch, low anti-nutrient content, high fiber content, and ability to flourish in low water stress situations. Lentil is cultivated in 52 countries; it spans an area of 3.85 million hectares and produce 3.59 million tonnes^15^. A very few researchers examined the impact of exogenously applied Si and nano-Si for reducing Pb in other crops but their effects with lentils is not widely studied yet. The study of lentils, particularly in the context of their physiological traits and biochemical activities, reveals findings regarding their potential to alleviate toxicity, especially in relation to heavy metals like arsenic and cadmium. However, the comparative studies to evaluate the effectiveness of bulk Si and nano-Si for reducing Pb concentration by leguminous lentils are very scarce.

Therefore, the current experiment has been planned with the objectives as: (1) to examine the impact of Si and nano-Si application for improving growth, yield and agro-physiological traits of leguminous lentils. (2) to evaluate the effect of Si and nano-Si application for reducing Pb by lentils.

## Materials and methods

### Experimental layout

A pot experiment was conducted to investigate the effect of bulk Si and nano-Si on promoting growth and reducing Pb concentration in lentil in a wire house having a glass covered roof (sides being open and only having iron wire screens with no control over temperature and humidity) at the Institute of Soil and Environmental Sciences (ISES), University of Agriculture Faisalabad (UAF), during the year 2022. The soil was obtained from the ISES UAF farm for air drying and sieving through a 2 mm sieve. Soil was analyzed for basic physico-chemical properties following the standard methods as described^16^. The soil was found sandy clay loam textured having pH of saturation paste (pH_s_) 7.91 electrical conductivity of saturated paste extract (EC_e_) 2.26 dS m^− 1^, organic matter 0.75% and ammonium-bicarbonate-diethylene-triamine-penta-acetic acid (AB-DTPA) extractable Pb in soil is 0.93 mg kg^− 1^. Each pot was filled with 8 kg soil and fertilizers dose of NPK at 20-40-20 kg ha^− 1^ was calculated for each pot and added to the processed soil. Next to preliminary analysis, the soil was spiked with Pb at 500 mg kg^− 1^ using lead nitrate as a source. Two levels of Si at 100 and 200 mg kg^− 1^ using sodium silicate as a source and one level of nano-Si at 125 mg kg^− 1^ was applied to soil for the alleviation of Pb toxicity by leguminous *Lens culinaris.* Lentil variety Punjab Masoor-2020 was tested and 5 seeds per plants were sown on November 15, 2022. The experiment was set up according to completely randomized design with three replications each.

### Preparation of nano-silicon

Nano-Si was synthesized by sol gel method as described^17^. In procedure, 18 ml sodium silicate was combined with 36 ml CH_3_COOH with 6.4 ml distilled H_2_O as a solvent. This procedure was carried out under continuous stirring. After that centrifugation of the resulting colloidal solution was done and then wash the centrifuged colloidal solution with 20 ml ethanol and then centrifuge again. The resultant precipitate was then oven dried for one day at 60 °C. After that, calcination will be done at the temperature of 600 °C for one and half hour to get the nano-silica powder. Then it was characterized by using Zeta analyzer. The nano-silica was characterized from National Institute for Biotechnology and Genetic Engineering (NIBGE), Faisalabad Pakistan. The average particle size was determined by zeta analyzer and for this purpose distilled water was used as dispersant medium. On the first day, forty milliliter of deionized H_2_O was combined with 0.1 g of sample and spun for 24 h. After 24 h, surface liquid (1 ml) was collected using a pipette and combined with 20 ml of deionized H_2_O for 24 h. After the 24 h, an average particle size study was performed. The sample preparation took three days to ensure that no agglomeration occurred and that single particle can be spotted.

### Measurement of physiological functions

The plants sampling and measurements for total chlorophyll contents (TCC), relative water contents (RWC) and membrane stability index (MSI) were conducted at the end of vegetative growth stage and the beginning of the reproductive phase of lentil plants.

Total chlorophyll contents of lentil leaves were measured at the completion vegetative growth stage by using Soil Plant Analysis Development (SPAD) meter-502 that works on the light emission and transmission principle. It is a low-cost mode to quantify plant photosynthetic capacity than expensive chlorophyll fluorescence and separate chlorophyll analysis via spectrophotometer. For determination of total chlorophyll contents three mature leaf of leguminous lentil plant were selected and placed under the SPAD meter and then average SPAD value of leaf was determined^18^.

Relative water content (RWC) is a measure of the physiological water status of a plant. For the measurement of RWC, young leaves from the lentil plants were taken to measure the RWC^19^. It is calculated using the following formula:

$${\text{RWC }}\left( \% \right){\text{ }} = {\text{ }}\left[ {\left( {{\text{Fresh weight }} - {\text{ Dry weight}}} \right){\text{ }}/{\text{ }}\left( {{\text{Turgid weight }} - {\text{ Dry weight}}} \right)} \right]{\text{ }} \times {\text{ 1}}00$$

Where:

Fresh weight is the weight of the leaf sample immediately after collection.

Dry weight is the weight of the leaf sample after drying in an oven.

Turgid weight is the weight of the leaf sample after rehydration in water for 24 h.

A higher RWC indicates better water status and abiotic (e.g., drought or heavy metal) tolerance of the plant.

The membrane stability index (MSI) measures the percentage of damage and describes the ability of the cell membrane to survive in drought stress conditions.

For the determination of MSI, young leaves from the lentil plants were taken to measure the MSI^20^. It is calculated using the following formula:

$${\text{MSI }}\left( \% \right){\text{ }} = {\text{ }}\left( {{\text{1 }} - {\text{ }}\left[ {{\text{C1}}/{\text{C2}}} \right]} \right){\text{ }} \times {\text{ 1}}00$$

Where:

C1 is the initial electrical conductivity of the leaf sample in deionized water after incubation at 40 °C for 30 min.

C2 is the final electrical conductivity after the sample is boiled at 100 °C for 10 min.

A higher MSI indicates greater membrane stability and tolerance to abiotic (e.g., drought or heavy metal) stress.

### Measurement of growth responses

Harvesting of lentil plants was done at maturity stage. Growth traits were determined by measuring shoot and root fresh weights, plant height, root length and grain yield at the time of harvesting. Initially samples were air dried and then kept the samples in an oven for 72 h to determine the shoot and root dry weights.

### Determination of pb concentration in shoot and root

Plants samples (shoot and root) of lentil were taken at harvesting, washed with sequentially tap water and distilled water to remove any adhering material. The plant materials were blotted dry with tissue paper and then air-dried for 2 days in the shade followed by oven-drying at 65 ± 5 °C for 72 h to obtain oven-dry weight. After oven-drying, the plant material was ground to a particle size < 1 mm using a mechanical grinder (MF 10 IKA, Werke, Germany). After grinding, wet digestion method was employed as the samples were uniformly mixed and 1-g portion was digested in a 3:1 mixture of nitric acid to perchloric acid at 150 °C^21^. Concentration of Pb in lentil shoot and root filtered digests was determined using flame atomic absorption spectrometry (FAAS; Model Thermo S-Series, Thermo Electron Corporation, Cambridge, UK)^22^.

### Statistical analysis

Statistics 8.1 software was employed to statistically observe data. The overall significance of data was assessed using the analysis of variance (ANOVA) method and LSD test at 5% level of significance. The *p* < 0.05 levels were found significant for differences among applied treatments^23^.

## Results

### Lentil physiological responses

**Fig. 1:**
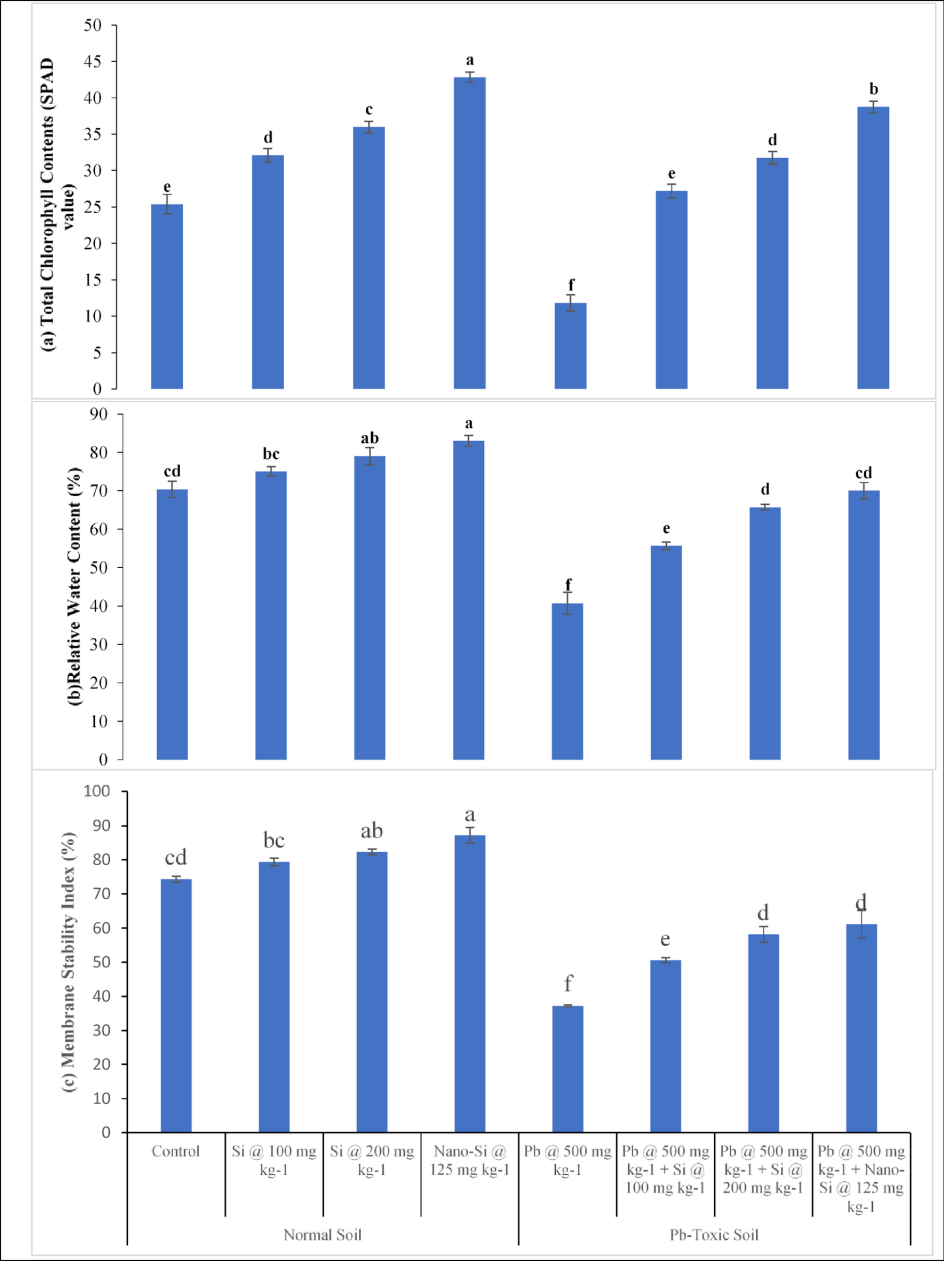
(**a–c**) Lens culinaris physiological responses (**a** Total chlorophyll contents (SPAD-value), **b** Relative water content %, **c** Membrane stability index %) as affected by applied Si and nano-Si in Pb-toxic soil condition (Means + SE, *n* = 3). [LSD values for treatments interactions: total chlorophyll contents, Pb × treatment = 2.5891; relative water content, Pb × treatment = 7.3009; membrane stability index, Pb × treatment = 7.2516].

The physiological parameter of lentil plants such as total chlorophyll contents (TCC, Fig. 1a), relative water content (RWC, Fig. 1b) and membrane stability index (MSI, Fig. 1c) were affected significantly (*p* ≤ 0.05, Table 1) by Pb, applied treatments and their interactive effects. The Pb toxicity in the soil resulted in reduction in TCC, RWC, and MSI of the plants. The Si and particularly nano-Si application increased TCC, RWC and MSI in both normal and Pb-toxic soil conditions. The Pb toxicity decreases TCC, RWC and MSI by 29%, 38.1% and 49.9% respectively as compared to respective controls. At control, application of Si at 100, 200 mg kg^− 1^ and nano-Si at 125 mg kg^− 1^ enhanced TCC by 26.5%, 41.7% and 68.7%, RWC by 6.6%, 12.3% and 18%, and MSI by 6.7%, 10.7% and 17.2% respectively. However, the Si application was effective in increasing the TCC, RWC and MSI in Pb-toxic soil conditions. At 500 mg kg^− 1^ Pb-toxic soil conditions, Si at 100, 200 mg kg^− 1^ and nano-Si at 125 mg kg^− 1^ enhanced TCC by 130.7%, 169.2% and 228.5%, RWC 36.9%, 61.5% and 72.1%, MSI 36%, 56.4% and 64.3% respectively, as contrast to respective controls. The nano-Si application at 125 mg kg^− 1^ was found to be more efficient in mitigating the harmful effect of Pb toxicity on the physiological parameters of lentil plants.

**Table 1:** F-values of two-way ANOVA for the *Lens culinaris* physiology, growth and tissue pb concentration as affected by applied Si and nano-Si in Pb-toxic soil condition.

Parameter	Pb	Si	Pb × Si
Total chlorophyll contents	113.81**	248.69**	11.58*
Relative water content	122.44**	29.12**	5.23*
Membrane stability index	300.26**	23.38**	3.37*
Plant height	15.76*	63.3**	3.65*
Shoot fresh weight	67.23**	110.76**	6.7*
shoot dry weight	70.82**	197.92**	7.06*
Root length	48.48**	77.78**	3.64*
Root fresh weight	60.26**	73.63**	4.38*
Root dry weight	77.82**	267.45**	5.17*
Number of pods	55.79**	127.96**	4.49*
Grain yield	69.63**	138.89**	5.97*
Pb concentration in shoot	265.93**	2556.35**	267.98**
Pb concentration in root	196.24**	2389.75**	198.01**

*NS* non-significant (*P* > 0.05); * = Significant (*P* ≤ 0.05); ** = Highly significant (*P* ≤ 0.01).

### Lentil growth responses

**Fig. 2:**
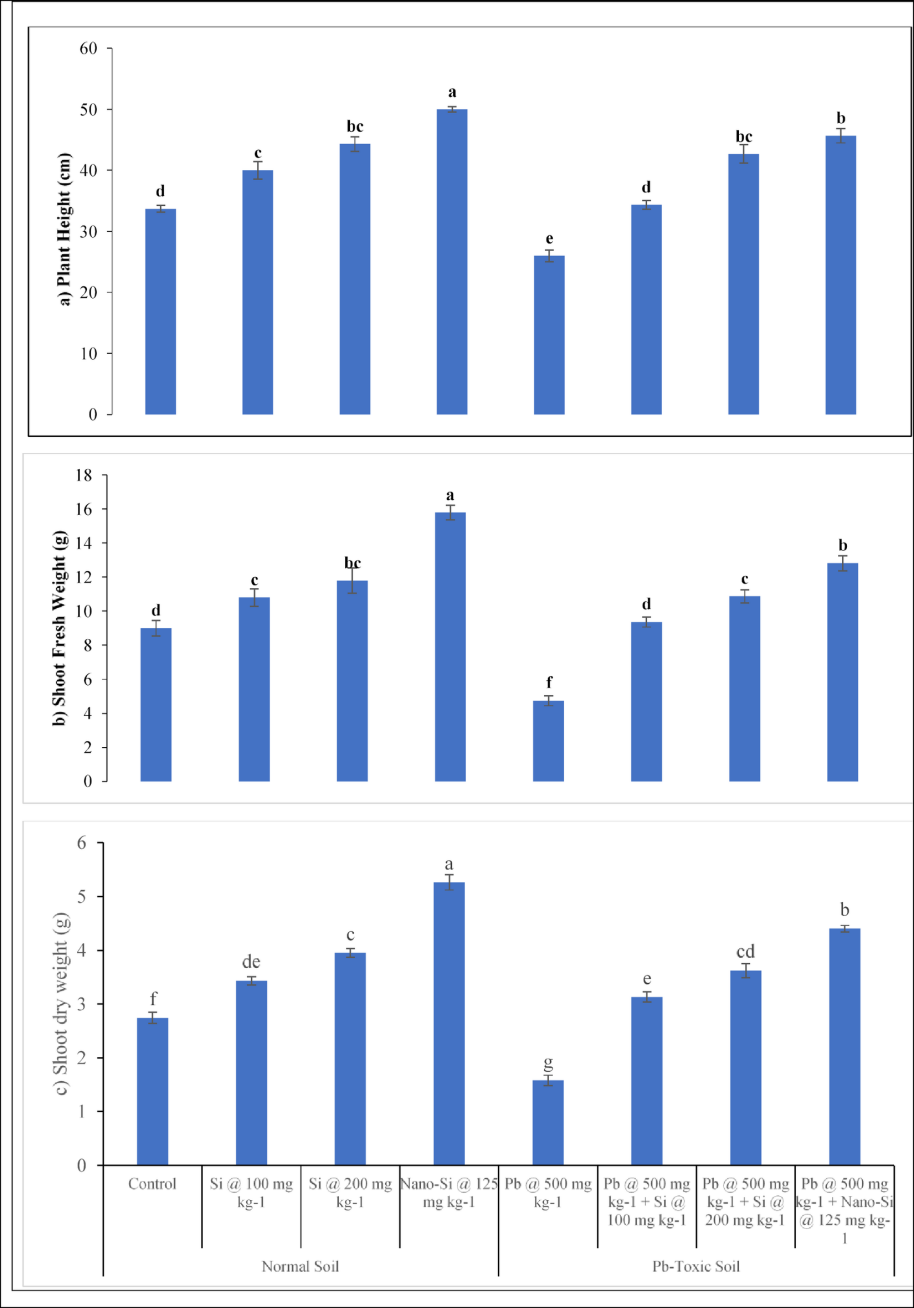

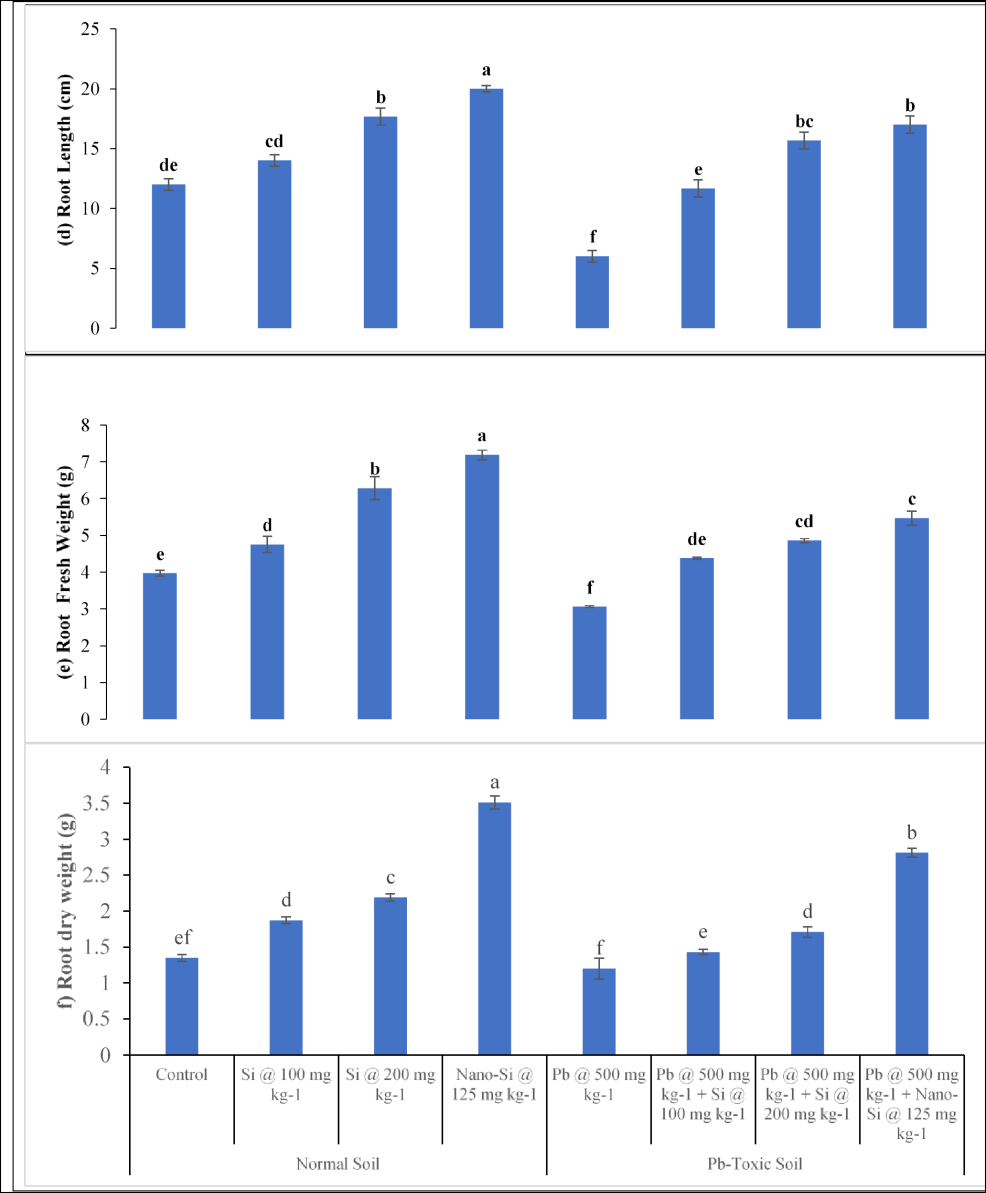
(**a–c**) Lens culinaris growth responses (**a** plant height, **b** shoot fresh weight, **c** shoot dry weight) as affected by applied Si and nano-Si in Pb-toxic soil conditions (Means + SE, *n* = 3). [LSD values for treatments interactions: plant height, Pb × treatment = 1.2517; shoot fresh weight, Pb × treatment = 1.2556; shoot dry weight, Pb × treatment = 0.3385]. (**d–f**). Lens culinaris growth responses (d = root length, e = root fresh weight, f = root dry weight) as affected by applied Si and nano-Si in Pb-toxic soil condition (Means + SE, *n* = 3). [LSD values for treatments interactions: root length, Pb × treatment = 2.2324; root fresh weight, Pb × treatment = 0.6120; root dry weight, Pb × treatment = 0.2144].

The lentil growth parameters including plant height (Fig. 2a), shoot fresh and dry weights (Fig. 2b and c), root length (Fig. 2d), as well as fresh and dry weight of root (Fig. 2e and f) were affected significantly (*p* ≤ 0.05, Table 1) by Pb, applied treatments and their interactive effects. Soil Pb toxicity noticeably decreased the plant height, root length, as well as fresh and dry weights for both the shoot and the root. The Pb toxicity decreases plant height by 22.7%, shoot fresh and dry weight by 47.3% and 42.3%, root fresh and dry weight by 22.9% and 11.1% and root length by 50% respectively as compared to respective control. In normal soils, application of Si at 100, 200 mg kg^− 1^ and nano-Si at 125 mg kg^− 1^ enhanced plant height (18.8%, 31.6% and 48.5%), shoot fresh (20%, 31% and 75.5%) and dry (25.1%, 44.1% and 91.9%) weight, root length (14.2%, 47.2% and 66.6%), root fresh (19.6%, 57.4% and 81.1%) and dry (38.5%, 62.2% and 160%) weights respectively. However, the Si application was efficient in increasing the plant height, root length, shoot and root fresh and dry weights in Pb-toxic soil. At 500 mg kg^− 1^ Pb-toxic soil conditions, Si at 100, 200 mg kg^− 1^ and nano-Si at 125 mg kg^− 1^ enhanced plant height (32%, 64.1% and 75.6%), shoot fresh (97.4%, 129.3% and 170.4%) and dry (98.1%, 129.1% and 178.4%) weights, root length (94.5%, 161.1% and 183.3%), root fresh (43.1%, 58.8% and 78.7%) and dry (19.1%, 42.5% and 134.1%) weights respectively, as compared to the respective controls. The nano-Si application at 125 mg kg^− 1^ was more efficient in mitigating the harmful effects of Pb toxicity on the growth of lentil.

#### Lentil yield responses

**Fig. 3:**
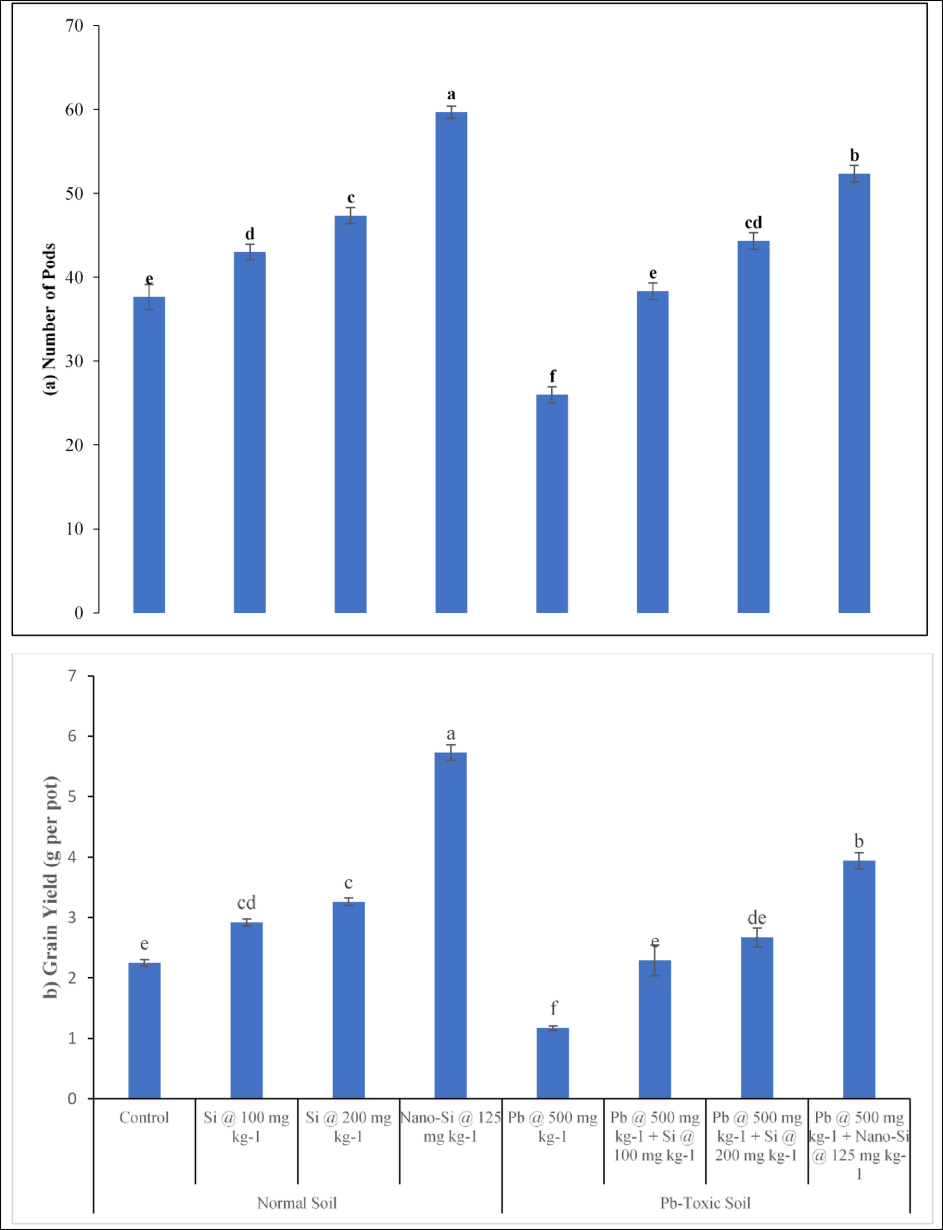
(**a,b**) Lens culinaris yield responses (**a** number of pods, **b** grain yield) as affected by applied Si and nano-Si in Pb-toxic soil condition (Means + SE, *n* = 3). [LSD values for treatments interactions: number of pods, Pb × treatment = 3.8286; Grain yield, Pb × treatment = 0.4939].

The lentil yield parameter including number of pods (Fig. 3a) and grain yield (Fig. 3b) were significantly affected (*p* ≤ 0.05, Table 1) by Pb, applied treatments and their interactive effects. Grain yield and number of pods were decreased as the Pb increased in soil. In comparison to control, Pb toxicity decreases grain yield and number of pods by 48% and 30.9% respectively. Under Pb toxicity at 500 mg kg^− 1^, application of Si at 100, 200 mg kg^− 1^ and nano-Si at 125 mg kg^− 1^ enhanced grain yield (29.7%, 44.8% and 154.6%) and number of pods (14.1%, 25.6% and 58.4%) respectively. However, the application of Si amendments was effective in increasing the number of pods and grain yield in Pb-toxic soil. At 500 mg kg^− 1^ Pb-toxic soil conditions, Si at 100, 200 mg kg^− 1^ and nano-Si at 125 mg kg^− 1^ enhanced number of pods (47.4%, 70.5% and 101.2%) and grain yield (95.7%, 128.2% and 236.7%) respectively, compared to respective controls. The nano-Si application at 125 mg kg^− 1^ was more efficient in mitigating the harmful effect of Pb toxicity on the lentil yield responses.

#### Pb concentration in root and shoot of lentil

**Fig. 4:**
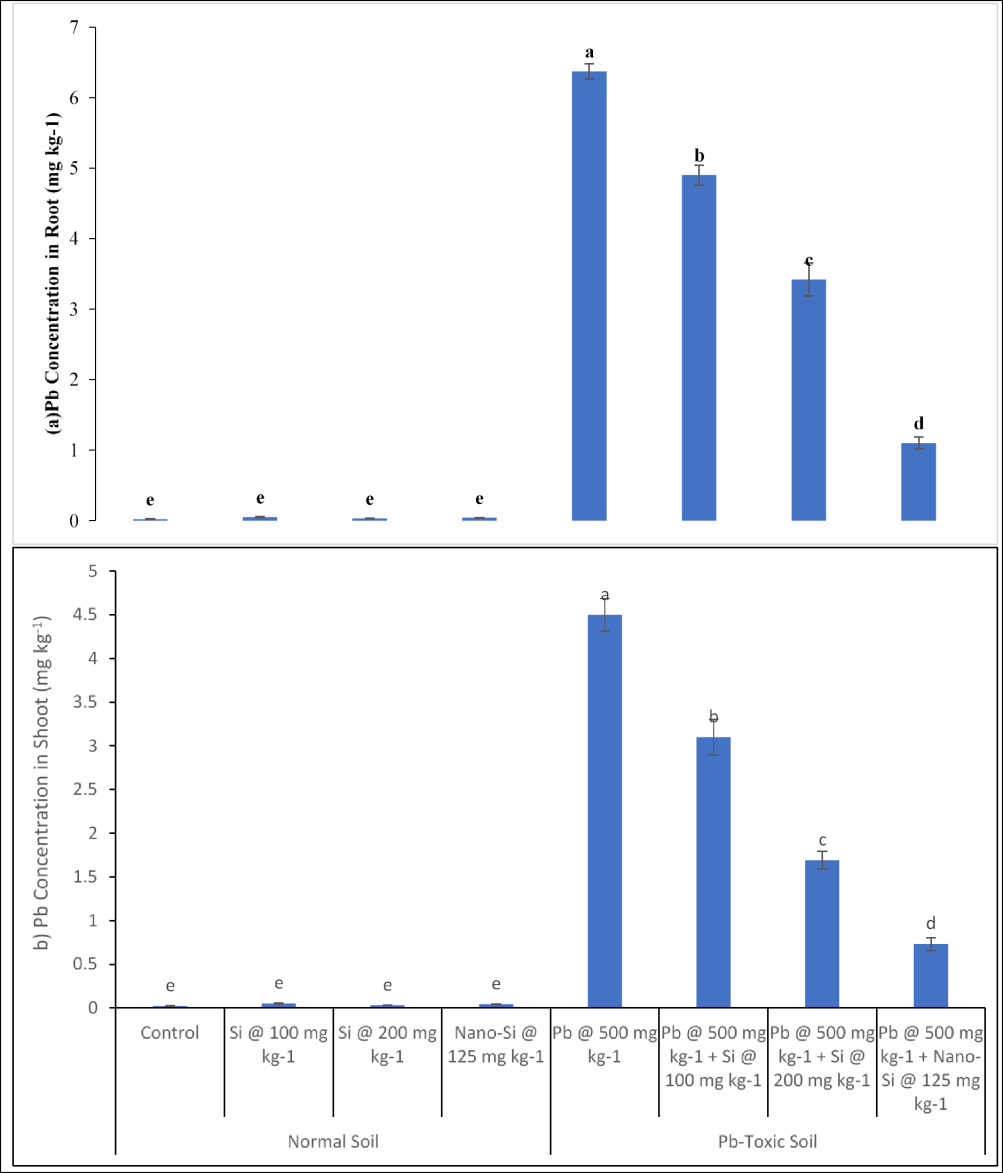
(**a,b**) Pb concentration in Lens culinaris (**a** Pb concentration in root,** b** Pb concentration in shoot) as affected by applied Si and nano-Si in Pb-toxic soil condition (Means + SE, *n* = 3). [LSD values for treatments interactions: Pb concentration in shoot, Pb × treatment = 0.2158; Pb concentration in root, Pb metal × treatment = 0.3422].

The Pb concentration in root (Fig. 4a) and shoot (Fig. 4b) of lentil are significantly affected (*p* ≤ 0.05, Table 1) by Pb, applied treatments and their interactive effects. The Pb toxicity increases Pb concentration in shoot and root by 224% and 317.5% respectively as compared to respective control. Under control, no Pb concentration in the root was noticed when there was no Pb in the soil. The maximum root Pb concentration was observed in 500 mg kg^− 1^ Pb when no Si amendment was applied. The minimum root Pb concentration was found at control. However, the Si application was efficient in decreasing the Pb concentration in shoot and root in Pb-toxic soil. At 500 mg kg^− 1^ Pb-toxic soil conditions, Si at 100, Si 200 mg kg^− 1^ and nano-Si at 125 mg kg^− 1^ decreased shoot Pb concentration (31.1%, 62.4% and 83.7%) and root Pb concentration (23%, 46.3% and 82.7%) respectively, relative to respective control. The nano-Si application at 125 mg kg^− 1^ was more efficient in minimizing damaging influence of Pb toxicity on the root and shoot Pb concentration of lentil.

## Discussion

In present study, lentil plant growth, physiological and biochemical characteristics were inhibited by Pb in Pb-toxic soil. An excess of Pb resulted in various toxic symptoms in plants including inhibited growth, chlorosis and darkening of the root system^24^. In these regards, phytoremediation can be a low-cost and very effective way of cleaning up the heavy metals contaminated soils. Leguminous plants may have the line of protection against metal translocation into biological cycles during the phytoextraction process. The main purpose of current experiment was to comprehend how Si and nano-Si increase the lentil growth, yield and decrease Pb absorption under Pb toxic soil conditions. Silicon is an element that can be applied to plants as a liquid or solid, and it has a substantial influence on development of plant roots, allowing faster growth and higher root resilience in problem soils. In addition, Si boosts plant’s immunity to resist abiotic and biotic stresses^6^. When Si is nurtured to crops, it has no negative impacts on the climate^8^.

In present study, total chlorophyll contents (Fig. 1a) were decreased drastically because of Pb toxic effects on the lentil plant. The Pb stress caused chlorosis and affected chlorophyll “a” more than chlorophyll “b”, which is the visible indications of Pb induced stress in crop plants that can be through alterations in chlorophyll levels, resulted in reduced photosynthetic efficiency^25^. The Pb stress reduced the chlorophyll contents in different crops inducing maize and rice owing to decreased availability of essential nutrients for the photosynthetic systems^25–27^. Heavy metal ions can bind to functional groups found in various enzymes (e.g., mercapto groups, -SH), displacing essential elements within these proteins. This type of substitution has potential to disrupt the formation of biological macromolecules and impede their functionality, ultimately leading to decline in photosynthetic capacity^28–30^. According to our pot trial findings, Si and particularly nano-Si application increased the total chlorophyll contents of lentils (Fig. 1a) grown under Pb stress. Silicon and nano-Si retains the ability to increase the chlorophyll contents in leaves by either enhancing the protective effects on photosynthetic pigments through improved leaf antioxidant activities or by activating processes that promote the production of these pigments^31^. Silicon has been observed to elevate the photosynthesis rate, as well as enhance chlorophyll content, delay the aging process of leaves, and raise the activity of the Rubisco enzyme^32^. Alternatively, Si can enhance the leaf chlorophyll index via a combination of these mechanisms. Furthermore, Si has the capacity to boost the chlorophyll levels in crop plants cultivated in recirculating nutrient solutions. Silicon application greatly boosts the chlorophyll contents of banana and maize^33,34^. The application of Si as Na_2_SiO_3_ enhanced the chlorophyll contents of wheat under stressful circumstances^35^.

The RWC (Fig. 1b) declined because of Pb toxic influence on leguminous lentil. The Pb ions interfere with transport of water to the aboveground parts of crop plants by preventing transpiration. Under Pb stress, transpiration is reduced whereas leaf thickness and size, intercellular gaps and stomatal pore size reduced. The stomata closure can be attributed to toxic metal stress either directly interacting with guard cells or owing to initial effects of metal poisoning on the roots and stems^36^. Heavy metals have a considerable influence on the gaseous exchange features of plants leaves, potentially leading to water stress because of impaired water transport from the soil to the leaves^37^. However, the application of Si and particularly nano-Si considerably decreased the detrimental effects of Pb toxicity in soil solution by improving absorption to leaf tissues, preserving vital nutrient transport from roots to plant leaves, and maintaining water holding capacity by leguminous crops. The Si application improves stomatal conductance and increases the water flow rate from roots to leaves^38^. The present study results positively correlated^39^ that applied Si-NPs significantly enhanced plant transpiration rate, stomatal conductance, MSI and RWC in heavy metal contaminated soil.

Plants exposed to Pb stress exhibited a significantly lowered the MSI (Fig. 1c) of lentil plants. Higher reactive oxygen species (ROS) reduce the MSI and increase cellular content leakage^40^. The decrease in cellular water content hampered the plants development and reduced the absorption of essential minerals by the cells. Consequently, both the size and number of cells decreased. The Pb effect the membrane stability by lowering cell membrane permeability, which causes a disruption in the cell’s ionic equilibrium^36^. Lead also brings changes in membrane working by modifying their lipid and fatty acid composition^41^. The Pb has been linked to an increase in ROS production in plants. The ROS oxidizes lipid membranes, reducing membrane stability^42^. Lead toxicity in wheat led to lower the MSI due to decreased cell wall elasticity^43^. Silicon serves as a protective barrier, decreasing cell membrane damage^44^. The Si and nano-Si treatment improved antioxidant activity and lowered leaf plasma membrane permeability, resulting in an increased MSI. Silicon improves cell membrane stability by avoiding damage to the plant cell membrane’s function, allowing it to preserve membrane stability and functionality under stressful condition^45^.

The lentil growth parameters such as plant height (Fig. 2a), shoot fresh weight (Fig. 2b), shoot dry weight (Fig. 2c), root length (Fig. 2d), root fresh weight (Fig. 2e), root dry weight (Fig. 2f) were reduced under Pb toxicity. The Pb toxicity is detrimental to plants growth and development because it prevents cells from dividing and expanding, therefore creating disturbances in physiological and biochemical processes which are needed for crop growth. Plants exhibited stimulation at low Pb concentrations but toxicity at high Pb concentrations^46^. The current experiment findings are in consonance with earlier research work demonstrating a substantial reduction in shoot and root biomass of legumes when cultivated in heavy metals contaminated soil^47^. The increased Pb in soil cause toxicity in brassica crops leading to decreased growth and physiological parameters^14^. A significant detrimental impact of Pb toxicity on root and shoot lengths, fresh and dry root and shoot weights of the cotton plants were also reported^48^.

The yield of lettuce plants was affected by Pb toxicity^46^. Increased Pb resulted in reduction of leguminous lentil yield parameters (Fig. 3a & b). In another study, the effect of Pb toxicity was found have very severe impacts on the roots and shoots of rice. With rise in Pb contents, the seed germination, root, shoot, and seedling growth, and dry biomass were significantly reduced^24^. The Pb exposure inhibited the root and shoot growth of *Brassica juncea*^49^. The root length was significantly decreased in *Brassica campestris* when Pb stress was induced^50^. In wheat, root length was adversely affected by increased Pb toxicity^51^ owing to significant rise in the Pb concentration of soils^52^. Another study established that plants readily absorb Pb from the soil, leading to its accumulation in various plant parts^53^. Silicon is deposited throughout the entire organism when the roots absorb Si from the soil and shift it to the shoot of plant through a transpiration stream^54^. Plant hormones, which are generated by certain organs and control a variety of growth and developmental processes, can be stimulated by the accumulated Si. In concord with previous research, the present study results also showed that applied Si at 200 mg kg^− 1^ and nano-Si at 125 mg kg^− 1^ is more effective in increasing yield parameters (Fig. 3a & b). Under Pb stress, Si supplementation protects plant tissues from membrane oxidative damage, lowering Pb toxicity. The present results positively correlated with exertion^55^ that plants grown with applied Si under metal showed considerably enhanced crop growth, fresh and dry masses, as well as leaf area.

The current study exhibited significant increase in Pb concentration in root (Fig. 4a) and shoot (Fig. 4b) under Pb toxicity. Findings from another study indicated that plants readily take up Pb from the soil and accumulates in various plant Sect^53^. The notable rises in the Pb content of cultivated soils were also reported^52^. The Si and nano-Si application lowered plant Pb concentration in shoots and roots by lentil. Silicon decreased Pb in shoots and roots as Si can create a metal-silicate complex with metals, which lowers metal mobility that make tolerance of toxic metals^56^. It has been identified that Si binds the metal and boost the plant’s tolerance and development^57^. The Si significantly improved Pb-affected cotton plant growth and decreased Pb accumulation in plants^48^. Silicon used to alleviate Pb contamination in *Solanum melongena L.* and make chelate under Pb toxicity^9^. Silicon reduced the impact of Pb concentration in lentil plants shoots. However, nano-Si at 125 mg kg^− 1^ significantly enhanced Pb affected plant growth, and reduced Pb accumulation in plant tissues of leguminous lentil. In inference, Si boosts metal tolerance in crop plants.

It has been reported that application of Si results in a rise in pH leading to reduced Pb availability^58,59^. Silicon forms Si-Pb complex and thereby precipitates Pb directly in the root^8,59^. Silicon reduced Pb translocation from root to shoot by effectively immobilizing Pb in plant^59^. Therefore, Si can create a Pb–silicate complex, which lowers Pb mobility and toxicity resulting in enhanced plant tolerance to Pb^56^. Silicon prevents Pb from reaching the cytoplasm and also enhances cell wall extensibility and encourages root elongation^57^. Silicon nanoparticles improve antioxidant enzymes activity including peroxidases, superoxide dismutase and catalase. The activity of peroxidases, superoxide dismutase and catalase was reported to enhance significantly due to the application of nano-Si as compared to the Pb stressed plants grown without the application of nano-Si^60^. This enhanced antioxidant capacity helped to neutralize ROS produced in plants in response to Pb stress, thereby decreasing the damage to plant cells caused by oxidative stress.

Our results indicated a reduction of Pb concentration in plants roots and shoots by the application of Si as bulk source and nanoparticles. However, the nano-Si were found more effective in reducing Pb concentration in tissues lentil plants (Fig. 4a & b). The application of nano-Si lowered Pb accumulation in both roots and shoots significantly under stress conditions^61^. The modifications in root structure and enhancement in the thickness of cell wall which served as a barrier against absorption of Pb were attributed as possible mechanisms for reduced concentration of Pb in plant tissues^62^. The heavy metal stabilization in soil by the incorporation of nano-silica is another mechanism by which Si reduces the mobility and availability of Pb in soil, and uptake and tissue Pb concentration in plants. The exchangeable Pb concentration in soil was reported to be reduced by the application of nano-Si in soil^63^. By improving soil structure and raising soil pH, nano-Si contributed Pb immobilization in soil, preventing its uptake by plants^63^.

Silicon has impacts on the expression level of metal transporters responsible for the absorption and translocation of heavy metal. A downward regulation of the expression of transporters facilitating the entry of Pb in plants has been reported due to the application of Si, thereby Si lowered the toxicity of Pb in plants^61,62^. This intonation is critical for sustaining cellular integrity in the presence of heavy metal stress. Silicon application especially in the form of nanoparticles offers a multidimensional approach for mitigating Pb toxicity in plants. Through improved antioxidant defenses, reduced metal uptake, immobilization of metals in soil, and modifying stress related gene expression, Si-NPs play an important role in supporting plant health under heavy metal stress conditions. The higher affectivity of Si nano-particles even at low concentration is due to their small structure which results in their higher solubility and better movement in plants leading to higher efficiency bulk Si sources are less soluble and their availability to plants is also low^64–66^.

## Conclusion

In present research work, bulk Si and nano-Si was used to decrease Pb concentration in lentils and enhancement in lentils growth and yield. The Pb-toxic soil conditions caused reduction in plant growth and development. However, Si and nano-Si application to soil minimized the concentration of Pb in plant tissues and improved the growth and key physiological characteristics. The Pb-toxic soil conditions negatively affected the growth and physiological traits of the lentil plants. However, the application of nano-Si at 125 mg kg^− 1^ showed the greatest outcomes by decreasing tissue Pb concentration resulting in reduced Pb toxicity to lentil plants prominent to better growth. To sum it up, the Si and particularly nano-Si certainly succeeded in reducing the Pb concentration and enhancing the growth and yield of lentil. The results of present study strongly support the use of Si and particularly nano-Si fertilizer recommendations for leguminous crops production under Pb-toxic soils.

## Data Availability

All data generated or analyzed during this study are included in this published article.
